# Supplementary material for: Anomalous time delays and quantum weak measurements in optical micro-resonators
Source: Nat Commun. 2016 Nov 14;7:13488. doi: 10.1038/ncomms13488 (PMC5476806; doi:10.1038/ncomms13488)
Supplement: Supplementary Information — Supplementary Figure 1, Supplementary Notes 1 & 2, and Supplementary References [file ncomms13488-s1.pdf]

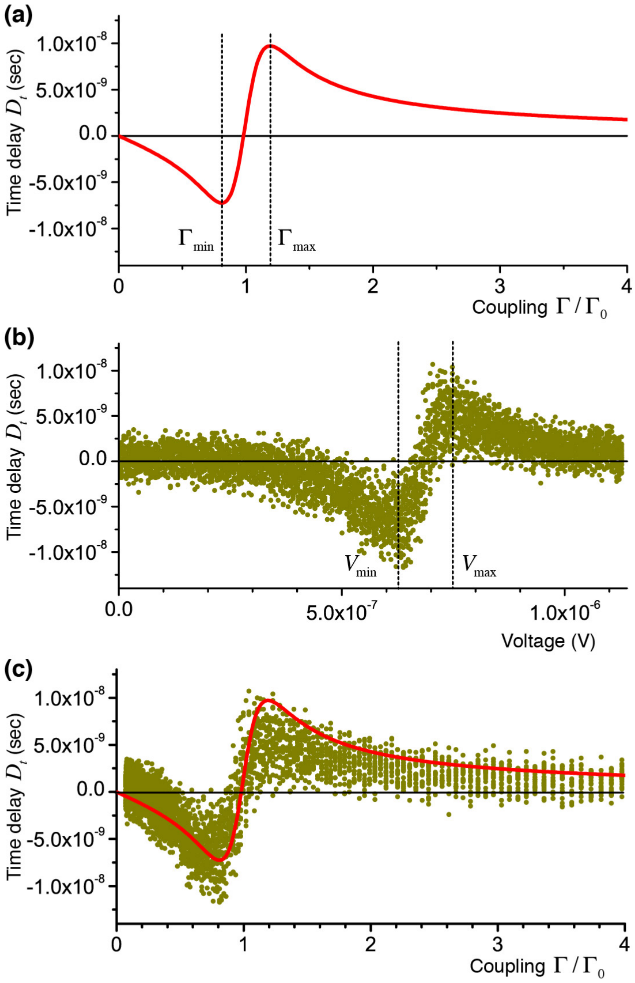


**Supplementary Figure 1** Determination of the coupling-parameter values and dependences (see explanations in the Supplementary Note 1).

## Supplementary Note 1. Determination of the coupling-parameter values and dependences from the experimental data.

The theoretical dependence of the time delay $D_t$ on the coupling parameter, $\Gamma$, has a resonant shape with two well-pronounced extrema (see Figure 3b and Supplementary Figure 1a). The experimentally-measured time delay as a function of the voltage $V$ of the positioner (changing the distance $d \propto V$ between the resonator and the fiber) also exhibits a similar resonant shape with two extrema (Supplementary Figure 1b).

As discussed in the main text, the relation between the voltage and coupling constant has the form $\Gamma = \alpha \exp(-\beta V)$ with two unknown constants $\alpha$ and $\beta$. Associating the voltages $V_{\mathrm{min}}$ and $V_{\mathrm{max}}$, corresponding to the extrema of the $D_t(V)$ curves, with the values $\Gamma_{\mathrm{min}}$ and $\Gamma_{\mathrm{max}}$, corresponding to the extrema in theoretical dependences $D_t(\Gamma)$, we retrieve the two parameters $\alpha$ and $\beta$. Finally, using equation $\Gamma = \alpha \exp(-\beta V)$, we plot the experimentally measured time delay $D_t$ versus the coupling strength $\Gamma$ (see Supplementary Figure 1c).

The above procedure was repeated for a series of measurements $D_t(V)$ with different detunings $\nu_{\mathrm{c}}$. Importantly, determining the constants $\alpha$ and $\beta$ at different detunings $\nu_{\mathrm{c}}$ resulted in approximately the same values (with variations ~10%). Therefore, we calculated the averaged values $\bar{\alpha}$ and $\bar{\beta}$ from all these series of measurements and used these values for the global mapping $\Gamma(V)$ in all the experimental data.

The final dependences of the time delays $D_t$ on the dimensional coupling parameter $\gamma / \Gamma_0 = (\Gamma - \Gamma_0) / \Gamma_0$ are shown in Figures 5d-f. We also used the obtained dependence $\Gamma(V)$ to determine the values of the coupling constant shown in the series of measurements with varying detuning, Figures 5a-c.

## Supplementary Note 2. Refined time-delay calculations.

The transmission coefficient $T(\omega)$ [Eq. (1) in the main text] connects the amplitudes of the Fourier components of the incident and transmitted fields, $\tilde{E}(\omega)$ and $\tilde{E}'(\omega)$. It is easy to see that in the time domain, the amplitudes of these signals, $E(t)$ and $E'(t)$, are connected by the differential equation

$$\frac{dE'}{dt}+(i\omega_0+\Gamma_0+\Gamma)E'=\frac{dE}{dt}+(i\omega_0+\Gamma_0-\Gamma)E. \tag{1}$$

The solution of this equation can be written in the integral form:

$$E'(t)=E(t)-2\Gamma\int_{-\infty}^{0} e^{(i\omega_0+\Gamma+\Gamma_0)\tau}E(t+\tau)d\tau. \tag{2}$$

The field of the incident wave packet can be written as $E(t)=\mathcal{E}(t)e^{-i\omega_c t}$, where $\mathcal{E}(t)$ is the slowly-varying amplitude. In a similar way, we write the transmitted wave-packet field as $E'(t)=\mathcal{E}'(t)e^{-i\omega_c t}$. In terms of these slow amplitudes, Eq. (2) becomes

$$\mathcal{E}'(t)=\mathcal{E}(t)-2\Gamma\int_{-\infty}^{0} e^{(-i\nu_c+\Gamma+\Gamma_0)\tau}\mathcal{E}(t+\tau)d\tau, \tag{3}$$

where $\nu_c=\omega_c-\omega_0$.

The typical scale of the temporal variations of the amplitude $\mathcal{E}(t)$ is assumed to be large as compared with the resonator relaxation time $(\Gamma+\Gamma_0)^{-1}\sim\Gamma_0^{-1}$, which is the adiabatic condition Eq. (3) or (14) in the main text. Then, one can expand $\mathcal{E}(t+\tau)$ in the Taylor series (keeping the *second*-derivative term)

$$\mathcal{E}(t+\tau)\simeq\mathcal{E}(t)+\tau\frac{d\mathcal{E}(t)}{dt}+\frac{\tau^2}{2}\frac{d^2\mathcal{E}(t)}{dt^2}. \tag{4}$$

Substituting Eq. (4) into Eq. (3), we evaluate the integral and arrive at

$$\mathcal{E}'(t)\simeq\frac{\nu_c-i(\Gamma-\Gamma_0)}{\nu_c+i(\Gamma+\Gamma_0)}\mathcal{E}(t)-\frac{2\Gamma}{\left[\nu_c+i(\Gamma+\Gamma_0)\right]^2}\frac{d\mathcal{E}(t)}{dt}+\frac{2i\Gamma}{\left[\nu_c+i(\Gamma+\Gamma_0)\right]^3}\frac{d^2\mathcal{E}(t)}{dt^2}. \tag{5}$$

Equation (5) is the time-domain analogue of Eq. (4) in the main text, but now keeping the second-derivative term in the Taylor series. It can be written in a compact form using the transmission coefficient [Eq. (1) in the main text] and its derivatives:

$$\mathcal{E}'(t)\simeq u_0\mathcal{E}(t)+iu_1\frac{d\mathcal{E}(t)}{dt}+\frac{i^2u_2}{2}\frac{d^2\mathcal{E}(t)}{dt^2}, \tag{6}$$

where $u_0=T(\omega_c)$, $u_1=\dfrac{dT(\omega_c)}{d\omega_c}$, and $u_2=\dfrac{d^2T(\omega_c)}{d\omega_c^2}$.

Let the temporal centroid of the incident wave packet be $t_c=\int_{-\infty}^{\infty}t|\mathcal{E}(t)|^2dt\Big/\int_{-\infty}^{\infty}|\mathcal{E}(t)|^2dt=0$. Then, the time delay of the transmitted wave packet is defined as

$$D_t = \frac{\int_{-\infty}^{\infty} t\left|\mathcal{E}'(t)\right|^2 dt}{\int_{-\infty}^{\infty}\left|\mathcal{E}'(t)\right|^2 dt}. \quad (7)$$

Assuming that the wave-packet envelope $\mathcal{E}(t)$ is real and symmetric with respect to $t = 0$, we evaluate Eq. (7) with Eq. (6). Cumbersome but straightforward calculations result in

$$D_t = \frac{\operatorname{Im}\left(u_0^* u_1\right) + \frac{1}{2}\operatorname{Im}\left(u_1^* u_2\right)\frac{I_1}{I_0}}{\left|u_0\right|^2 + \left[\left|u_1\right|^2 + \operatorname{Re}\left(u_0^* u_2\right)\right]\frac{I_1}{I_0}}, \quad (8)$$

where $I_0 = \int_{-\infty}^{\infty}\left|\mathcal{E}(t)\right|^2 dt$ and $I_1 = \int_{-\infty}^{\infty}\left|d\mathcal{E}(t)/dt\right|^2 dt$. For the Gaussian incident pulse, Eq. (2) in the main text, we have $\mathcal{E}(t) \propto \exp\left(-t^2/2\Delta^2\right)$ and $I_1/I_0 = \tilde{\Delta}^2/2$.

If we neglect the second-derivative terms in Eq. (S8), $u_2 \to 0$, it becomes equivalent to Eqs. (11) and (12) in the main text. With the $u_2$ terms, Eq. (8) represents a 1D temporal analogue of the 2D beam-shift equation derived in [1]. When the adiabatic parameter $\varepsilon = \tilde{\Delta}/\Gamma_0$ is sufficiently small, the $u_2$-terms practically do not affect the $D_t(\nu_c, \gamma)$ dependences, and could be safely neglected (see Figure 5).

One can also note that the integral in Eq. (3) can be evaluated exactly for the Gaussian incident pulse. This yields

$$\mathcal{E}'(t) = \mathcal{E}(t)\left\{1 - \sqrt{2\pi}\,\Delta\Gamma e^{z^2}\left[1 - \operatorname{erf}(z)\right]\right\}, \quad (9)$$

where $z = \left[\Delta\left(\Gamma_0 + \Gamma - i\nu_c\right) - \Delta^{-1}t\right]$. Equation (9) allows calculation of the time and frequency shifts even when the adiabatic parameter $\varepsilon$ is not small. However, in this case, the spectrum width of the pulse becomes of the order of or wider than the resonator linewidth, and the approximate resonance-transmission equation (1) in the main text can become invalid for side frequencies with $\left|\omega - \omega_0\right| \gg \left(\Gamma_0 + \Gamma\right)$.

## Supplementary References


1. J.B. Götte and M.R. Dennis, “Limits to superweak amplification of beam shifts,” *Opt. Lett.* **38**, 2295–2297 (2013).
